# Theoretical Basis, Laboratory Evidence, and Clinical Research of Chemical Surgery of the Cornea: Cross-Linking

**DOI:** 10.1155/2014/890823

**Published:** 2014-08-18

**Authors:** Amanda C. da Paz, Patrícia A. Bersanetti, Marcella Q. Salomão, Renato Ambrósio, Paulo Schor

**Affiliations:** ^1^Department of Ophthalmology and Visual Sciences, Paulista School of Medicine, Federal University of São Paulo (UNIFESP), São Paulo, SP, Brazil; ^2^Department of Health Informatics, Paulista School of Medicine, Federal University of São Paulo (UNIFESP), 04023-062 São Paulo, SP, Brazil; ^3^Instituto de Olhos Renato Ambrósio, 20520-050 Rio de Janeiro, RJ, Brazil

## Abstract

Corneal cross-linking (CXL) is increasingly performed in ophthalmology with high success rates for progressive keratoconus and other types of ectasia. Despite being an established procedure, some molecular and clinical aspects still require additional studies. This review presents a critical analysis of some established topics and others that are still controversial. In addition, this review examines new technologies and techniques (transepithelial and ultrafast CXL), uses of corneal CXL including natural products and biomolecules as CXL promoters, and evidence for *in vitro* and *in vivo* indirect effectiveness.

## 1. History

The concept of using collagen cross-linking photochemically induced, for increasing corneal stiffness, as a conservative method to stabilize ectasia progression was first conceived in Germany in the 1990s by Theo Seiler and collaborators [1–4]. Collagen cross-linking (CXL) opened a new horizon for conscious biomechanical manipulation of the cornea [5], which uses the concept of biomechanical customization of therapeutic and refractive corneal surgery [6]. The original “Dresden cross-linking clinical protocol” involves topical anesthesia, central corneal abrasion, and application of riboflavin 0.1% with 20% dextran T-500 until stromal saturation is observed through biomicroscopy. The traditional procedure is followed by ultraviolet A (UVA) light of 365–370 nm at an irradiance of 3 mW/cm^2^, which corresponds to a dose of 5.4 J/cm^2^ for 30 min [3].

The photopolymerization effect on corneal collagen results from the reaction of the photosensitizer agent riboflavin and UVA light (370 nm), which is the absorptive peak of riboflavin. This reaction generates reactive oxygen species that can react with various molecules and subsequently induce chemical covalent bonds that bridge the amino groups of collagen fibrils [7]. Hayes et al. (2013) demonstrated riboflavin/UVA-induced cross-links at the surface of the collagen fibrils and within the proteoglycan (PG) rich coating surrounding them [8]. In another study, Zhang et al. (2011) reported that riboflavin/UVA treatment causes beyond CXL among collagen molecules and among PG core proteins, as well as limited linkages between collagen and PG such as mimecan, decorin, keratocan, and lumican [9].

## 2. Other Approaches

### 2.1. Natural Cross-Linking

Human collagen undergoes progressive changes including a decrease in solubility, elasticity, and permeability, as well as an increase in thermal stability and resistance to enzymatic digestion with aging. The precise chemical changes of these transformations are unknown. However, an* in vitro* study has suggested that these physical changes involve progressive CXL among collagen molecules [10]. A detailed study of the collagen fibrils in normal human corneas showed a small but significant age-related increase in collagen fibrils for diameter, intermolecular spacing, and elongation [11]. Expansion of the collagen intermolecular spacing suggests molecules other than collagen are deposited between the fibrils during aging, which subsequently push the collagen molecules further apart. This is consistent with a recent study that demonstrated glycation-induced expansion of intermolecular spacing and subsequent CXL of molecules with age [10]. Considering the isolated ultrastructural dimensions of collagen fibrils, one would expect a tendency toward biomechanical strengthening of the cornea during aging [12].

Hyperglycemia was shown to influence corneal biomechanical properties by inducing stromal collagen CXL through glycosylation and lysyl oxidase (LOX) enzymatic activity [13]. People with diabetes mellitus have increased central corneal thickness, corneal hysteresis, and a corneal resistance factor, possibly reflecting a greater stiffness of diabetic corneas [14].

### 2.2. Biomolecules and Natural Products

Several studies have demonstrated several molecules that might promote collagen cross-link. Natural products such as genipin [15, 16] and proanthocyanidins (PAs) [17] can form cross-links between collagen fibrils. Avila et al. (2012) demonstrated in an* ex vivo* study that corneal CXL with genipin was similar to the UV traditional procedure, with minimal toxicity to endothelial cells [16]. PAs are natural products with polyphenolic structures that have the potential to give rise to stable hydrogen bonded structures and generate nonbiodegradable collagen matrices. Han et al. (2003) demonstrated the feasibility of using PAs from grape seeds to cross-link collagenous materials [17].

Biomolecules, such as the leucine-rich proteoglycans (e.g., decorin, lumican, and keratocan), regulate the orderly assembly of extracellular matrices, corneal transparency, tensile strength of skin and tendons, viscoelasticity of blood vessels, and tumor cell proliferation. Experiments* in vitro* showed that SLRPs interact with collagen through specific binding sites and delay formation of collagen fibrils. To modulate cornea collagen fibrillogenesis decorin binds to collagen types I, II, III, VI, and XIV [18, 19].

## 3. *In Vitro* Effectiveness Evidences

### 3.1. Increase in Collagen Fiber Diameter

Riboflavin/UVA-induced collagen CXL increases the corneal collagen fiber diameter, which was more pronounced in the anterior portion of the stroma of the rabbit cornea as observed on transmission electron microscopy [23].

### 3.2. Resistance to Enzymatic Digestion

The stabilizing biochemical effect of CXL can be explained by changes in the tertiary structure of collagen fibrils induced by CXL preventing access of the proteolytic enzymes to their specific cleavage sites by steric hindrance. In porcine corneas cross-linked with riboflavin/UVA, CXL causes an impressive doubling in the time following pepsin, trypsin, and collagenase digestion, particularly in the anterior half of the cornea [22].

### 3.3. Modulus of Elasticity (Young's Modulus)

Many published studies report an increase in cornea stiffness after collagen CXL. Wollensak et al. (2003) found a significant increase in biomechanical rigidity by a factor of 4.5 in human corneas following riboflavin/UVA-induced collagen CXL, which was indicated by an increase in Young's modulus. The increase in biomechanical stiffness in porcine eyes was also significant by a factor of 1.8 [20]. In another study, Wollensak and Iomdina (2009) found a highly significant increase in corneal stiffness after CXL treatment of rabbit corneas with an impressive durability over time, as demonstrated by a 78.4%–87.4% (by a factor of 1.6) increase in Young's modulus by and a 69.7%–106.0% increase in ultimate stress over the entire 8-month follow-up [21]. Some limitations of this method are that the strip specimens originated from a curved sample, the corneal structure is disrupted because the lamellae are cut, and several crucial constraints are ignored (e.g., real pachymetry and meridional differences) [31].

### 3.4. Atomic Force Microscopy

Atomic force microscopy (AFM) has a shaping probe tip that can scan the sample surface at an atomic distance. By monitoring the interaction force between the tip and the sample surface, this instrument can create topographical images of the sample surface at high resolutions [32]. When the probes approach the sample surface, tiny interaction forces, such as Van der Waals and electrostatic forces, occur between the probe and sample. The resulting cantilever is recorded by measuring the displacement of a laser beam reflected from the backside of the cantilever. AFM can be applied to identify the collagen bundles and to determine their diameters [33]. This technique provides quantitative information on the surface morphology of the collagen fibrils at a high resolution [32]. Yamamoto et al. (2002) clearly obtained surface topographic images of human corneal and scleral collagen fibrils using AFM [32]. Further AFM studies are important to examine cross-link induced modification in corneal collagen fibrils. Seifert et al. (2014) developed a method that allows for atomic force microscopy-based measurements of gradients of Young's modulus in soft tissues. In the abovementioned study, the authors demonstrated the depth-dependent distribution of the stiffening effect caused by riboflavin/UV CXL in porcine corneas [34].

### 3.5. X-Ray Scattering

X-ray scattering is a specialized technique that provides structural information about the constituent collagen in the corneal stroma. The wide-angle equatorial scattering pattern produced from the lateral packing of molecules within the stromal collagen fibrils can be used to determine the intermolecular spacing within the fibrils, as well as the arrangement and distribution of fibrillar collagen in the intact cornea [35, 36]. X-ray scattering is a unique method for measuring the lateral space between individual fibril-forming collagen molecules at less than a 1 mm resolution. This space is influenced by both the fibril hydration and the extent of molecular CXL [35]. Studies of corneal collagen organization in keratoconus (KC) suggest that the mechanism of tissue thinning in this disease involves fibrillar or lamellar collagen slippage, decreased lamellar interweaving [35, 37], and distortion of the orthogonal matrix [37]. The authors of study proposed that development of interventional cross-linking strategies may limit collagen slippage and should be beneficial for delaying the progression of keratoconus [35, 37]. In another study that analyzed CXL in human corneas using X-ray scattering, Hayes et al. (2011) concluded that UVA/riboflavin induced cross-links do not have a measurable effect on the axial stagger or the tilt of collagen molecules within the fibrils when analyzed using X-ray scattering method [36].

### 3.6. Second Harmonic Generation Microscopy

Second harmonic generation (SHG) microscopy has been used extensively in medicine and biology to obtain images of highly ordered structures, such as collagen fibers, microtubulin, and skeletal muscle, with high resolution and contrast. This nonlinear optical microscopy results from a coherent second-order nonlinear scattering wherein a noncentrosymmetric structure emits light at half the wavelength of the incident (pump) optical field. Collagen fibers, being intrinsically noncentrosymmetric, emit SHG and thus produce high-contrast images without the need for staining [38].

Collagen fibrils are aligned uniformly in the corneal stroma and are therefore believed to be responsible for SHG from the cornea. SHG imaging has thus allowed visualization of collagen organization and can be processed to generate three-dimensional reconstructions of collagen structure [39].

In 12 of 13 human keratoconic corneal samples obtained after penetrating keratoplasty for KC, SHG could detect differences in the organizational pattern of lamellae, including a marked loss or decrease in anterior lamellae interweaving and lamellae that inserted into Bowman's layers [40].

Analysis of porcine corneas with and without riboflavin/UVA CXL treatment using SHG showed that stromal collagen fibrils in untreated corneas had a more regular, linear, and parallel orientation. However, treated corneas had wavy stromal collagen fibrils [41].

## 4. *In Vivo* Indirect Effective Evidences

### 4.1. Visual Acuity

The primary goal of CXL is to improve the biomechanical rigidity of corneal collagen to stop ectasia progression [1, 2]. In the first published clinical trial, Wollensak et al. (2003) reported stability after CXL treatment of the eyes of 19 patients with progressive KC and with a mean follow-up of 20 months (from 3 to 33 months) [3]. In this series, visual acuity (VA) slightly improved in 15 eyes (65%). The improved uncorrected visual acuity (UCVA) recorded during the follow-up is partially explained by the sphere and spherical equivalent reduction. However, these data also may be related to a progressive reduction of the mean K power. Furthermore, the increased best spectacle-corrected visual acuity (BSCVA) may be linked to a reduction in the difference between superior and inferior corneal hemimeridians (flattest versus steeper), expressed by the improvement in corneal symmetry indexes. Moreover, an increased BSCVA may be sustained by the statistically significant early reduction in coma aberration [42].

### 4.2. Keratometry

In the first published clinical trial [3], there was a variable disease regression observed in 16 cases (70%) by a reduction of the maximal keratometry readings and refractive error [3]. Similar results were observed in other studies examining CXL for KC [43–50] and keratectasia [48, 51–54]. Corneal reshaping [55] appears to be a more reliable expression of CXL induced clinical and topographic changes. Mean clinical and topographic improvements were recorded from the end of the third postoperative month and continued thereafter, reaching reliable stability in 24 months [46]. In addition, Koller et al. (2009) found KMax to be an important prognostic variable, which was associated with a significant reduction in complications when excluding cases with a KMax higher than 58D [56]. A higher chance of ectasia regression, observed by flattening, was more likely if KMax was higher than 54D [57].

### 4.3. Biomicroscopy

A stromal demarcation line, biomicroscopically detectable as early as 2 weeks after CXL treatment, was described by Seiler and Hafezi (2006) as the first clinical evidence of a physical effect of CXL on corneal tissue [58]. The demarcation line does not refer to biomechanical properties but represents the transition between cross-linked anterior corneal stroma, with modified refractive and reflection properties, and the untreated posterior corneal stroma [58]. Caporossi et al. (2010) found stromal edema, clinically detectable by slit-lamp examination in 70% of patients, occurred in the first 30 postoperative days. Temporary haze occurred in 9.8% of cases, 14 cases in the first 3 months, and 2 cases after 6 months but disappeared progressively after topical preservative-free steroid therapy [46].

### 4.4. Scheimpflug Photography and Optical Coherence Tomography

The stromal demarcation line is also observed via Scheimpflug photography [59–62] and optical coherence tomography (OCT) [46, 63]. Visante OCT scans show a higher reflectivity (hyperdensity) of this line, and after 6 months, stromal reflectivity becomes more homogeneous, reducing the visibility of the line in some eyes much more than in others [46].

### 4.5. Pachymetry

The pachymetric map provides the thinnest point data, which is critical for ensuring the safety parameters for the endothelium [64]. The thickness map also should be important for monitoring results after CXL. Corneal thinning has been documented in the early CXL postoperative course, with a gradual return on corneal thickness toward preoperative values within the first year after CXL [45, 46, 62, 65].

### 4.6. Ocular Response Analyzer

Until the launching of the ocular response analyzer (ORA) (Reichert Inc., Depew, NY) in 2005 [66], corneal biomechanical studies were limited to laboratory* in vitro* studies and virtual mathematical corneal finite element models [67, 68]. ORA is a modified noncontact tonometer (NCT) that was designed to provide a more accurate measurement of IOP through an understanding of compensation for corneal properties [66].

During an ORA measurement, a precisely metered air pulse is delivered to the eye, causing the cornea to move inward, past a first applanation (flattening), and into a slight concavity. Milliseconds after the first applanation, the air pump generating the air pulse is shut down and the pressure applied to the eye decreases in an inverse-time symmetrical fashion. As the pressure decreases, the cornea passes through a second applanated state while returning from concavity to its normal convex curvature [66].

An electrooptical collimation detector system monitors the corneal curvature in the central 3.0 mm diameter throughout the 20-millisecond measurement. A filtered (smoothed) version of the detector signal defines 2 precise applanation times corresponding to 2 well-defined peaks produced by inward and outward applanation events. Two corresponding pressures of an internal air supply plenum are determined from the applanation times derived from the detector applanation peaks [66].

The system registers the independent applanation pressures during the ingoing (P1) and outgoing (P2) phases. The difference between the 2 pressures is called corneal hysteresis (CH) [69, 70]. Corneal resistance factor (CRF) is also calculated from P1 and P2 with an optimized function designed to augment the correlation with thickness in a normal population [66, 70]. CH and CRF were significantly lower in keratoconus, but CH and CRF were unchanged after CXL [71–73]. Hysteresis is a viscoelastic property of the cornea that is not directly related to stiffness [74]. A new set of parameters derived from the waveform ORA signal that monitors the deformation response of the cornea during an ORA measurement has been reported [72–76]. These parameters had a better diagnostic performance for keratoconus [75, 76]and improved after CXL [74, 76].

### 4.7. Corvis

Corvis has an ergonomic design. The patient is comfortably positioned with proper placement of the chin and forehead and then asked to focus on a central red LED. A frontal view camera is mounted with a keratometer-type projection system for focusing and aligning the corneal apex. The examination is programmed for automatic release when alignment is achieved with the first Purkinje reflex of the cornea [77].

This equipment is a NCT system integrated with an ultrahigh speed (UHS) Scheimpflug camera that was introduced by Ambrósio Jr et al. (2013) [77]. The CorVis ST (Scheimpflug Technology) records 4,330 frames per second, with a Scheimpflug camera that covers 8 mm horizontally, to monitor the corneal response to a fixed profile air pulse with a maximal internal pump pressure of 25 kPa. The addition of an UHS Scheimpflug camera allows dynamic inspection of the actual deformation process that provides further details for biomechanical characterization of the cornea.

The recording starts with the cornea at the natural convex shape. The air puff forces the cornea inward (ingoing phase) through applanation (first or ingoing applanation) into a concavity phase until it achieves the highest concavity (HC). Thereafter, the cornea undergoes a second applanation before achieving its natural shape [77]. The parameters derived from the corneal response such as corneal speed during deformation, corneal applanation length, deformation amplitude (greatest displacement of the apex at the point of HC), and radius of curvature at HC are important measures of corneal viscoelastic properties and stiffness. Such parameters are useful for the diagnosis of ectasia [75] and assessing CXL results.

In an ancillary study conducted at the Ohio State University in an industry-sponsored FDA trial of corneal collagen CXL, subjects were evaluated biomechanically using the CorVis ST before and after the procedure. Preliminary analysis at 1-month postprocedure was performed with 11 keratoconic subjects randomly selected for treatment, compared with 8 keratoconic subjects randomly selected for the sham group. A significant difference (*P* < 0.0014) was found in the radius of curvature at HC in subjects who received treatment, which is consistent with increased stiffness. Subjects in the sham group showed no difference (*P* = 0.6981) at 1 month [77].

### 4.8. Confocal Microscopy

*In vivo* confocal analysis showed disappearance of keratocytes in the anterior midstroma to a depth of 340 *μ*m [55] and a clear vertical transition area between the edematous hyporeflective stroma with apoptotic bodies and normoreflective deep stroma. After 6 months, the reflectivity of the anterior midstroma was inverted (hyper) compared with initial postoperative reflective previously demonstrated [55]. Changes in the stromal reflectivity after the sixth month are an important indirect (confocal) sign of corneal CXL [55]. In general, after the third month, there is new collagen synthesis meditated by repopulating keratocytes and lamellar compaction, expressed by the hyperreflectiveness of the extracellular matrix, combined with newly formed collagen fibers identified with* in vivo* confocal scans [55, 78]. In addition to this finding, nerve plexus degeneration was noted up to 6 months postoperatively following CXL [79].

Confocal microscopy demonstrated numerous hyperreflective spherical structures more abundantly in the anterior stroma, and they were visible up to a depth of 300 *μ*m after CXL [80]. It is not clear what these structures represent; however, they may represent damaged keratocytes or nuclear and cellular fragments. The stroma had a spiculated appearance and extended to a depth at 300 *μ*m that could be secondary to changes in stromal hydration [80].

The increase of collagen fiber diameter could partly explain the increased scattering of the collagen fibers creating a net-like formation observed at the first and third months after CXL [81]. In addition, revelation of the otherwise unseen collagen fibers in the confocal microscopy images also suggest alterations of the normal collagen fiber formation that is responsible for the transparency of the cornea in normal conditions. This may also have implications on the vision function and contrast sensitivity [81].

## 5. Another Crosslinking Protocols

### 5.1. Transepithelial Cross-Linking (Epithelial Damage versus Amphiphilic Molecules)

Analysis of the light transmission spectra of porcine corneas following riboflavin/UVA corneal CXL treatment suggests a need for completely removing the epithelium to allow adequate and homogeneous penetration of riboflavin into the stroma [82]. A grid pattern of full thickness epithelial debridement appears to allow some riboflavin stromal penetration; however, this was less significant compared with that observed after complete central epithelium removal [82]. An application of 20% alcohol in the presence of an intact epithelium is not sufficient to allow adequate riboflavin penetration into the corneal stroma [82]. A riboflavin complex with ethylenediaminetetraacetic acid (EDTA) and trometamol was used for transepithelial CXL after superficial scraping. However, the uptake was considerably less than in corneas with epithelium removed [83]. Pharmacological permeabilization of epithelium was achieved by applying cyclodextrins that enhance riboflavin solubility in water and to improve its permeability through bovine corneas [24]. Raiskup et al. (2012) showed that a riboflavin solution without dextran, but including 0.01% benzalkonium chloride and 0.44% NaCl promoted the permeability through the epithelium, resulting in a sufficient concentration of riboflavin in the stroma [25]. Recently, Bottos et al. (2013) described riboflavin nanoemulsions that could penetrate the corneal epithelium. A greater stromal concentration was detected after 240 min when compared with corneas submitted to the standard protocol [84]. Bikbova and Bikbov (2014) showed the effectiveness of the impregnation of riboflavin 0.1% in eyes of 19 patients by iontophoresis in transepithelial collagen CXL with a decrease in the average keratometry 1 year after the procedure [85].

### 5.2. Athens Protocol

Kanellopoulus et al. (2009) studied topography-guided PRK at least 6 months following CXL and topography PRK followed immediately by CXL in a single procedure in adults with advancing KC to stabilize ectasia and rehabilitate vision (with topography-guided PRK) [30]. The simultaneous procedure appeared to be superior to sequential treatments in rehabilitation of keratoconus with minimal haze formation, and in addition to a reduction in the patient's time away from work. Perhaps CXL will have a wider application as prophylaxis in laser refractive surgery [30]. In another study, the same author found potentially promising results with the same-day and simultaneous topography-guided PRK and collagen CXL as a therapeutic intervention in highly irregular corneas with progressive corneal ectasia after LASIK [86].

### 5.3. Ultrafast Cross-Linking

According to the Bunsen and Roscoe (1862) law, the effect of a photochemical or photobiological reaction is directly proportional to the total irradiation dose, irrespective of the time span over which the dose is administered [87]. Schumacher et al. (2011) found an increase in Young's modulus statistically equivalent in the group of porcine corneas treated with illumination intensity of 10 mW/cm^2^ and 3 times shorter illumination time of 9 min compared with a group with an intensity of 3 mW/cm^2^ that required an illumination time of 30 min [27]. High fluence and UV light used with shorter exposure appears to be safe and effective in stabilizing keratoconus, and this technique appears to be similar but more comfortable for patients [28].

In Table 1 are showed* in vitro* and* in vivo* evidences of Dresden protocol and the new approaches of CXL.

## 6. Conclusions

Clinical assessment of biomechanical properties represents an area of active research. Novel nondestructive methodologies have been described, including radial shearing speckle pattern interferometry [88, 89], Brillouin optical microscopy [90], and other forms of dynamic corneal imaging [91, 92]. These approaches may soon be developed into commercially available instruments.

CXL has revolutionized the treatment of ectatic diseases. However, considering the goal of the procedure is to stiffen corneal tissue, thereby stabilizing ectasia progression, characterization of the cornea should go beyond shape analysis into biomechanical assessment. Such characterization is critical for enabling conscious optimization and further improvements in CXL techniques. Such advances should significantly affect the indication, planning, and postoperative evaluation of ectasia treatments.

## Figures and Tables

**Table 1:** *In vitro* and *in vivo* evidences of corneal cross-linking protocols.

Protocol	*In vitro *	*In vivo *
Epi-off CXL (Dresden Protocol)	Increased Young's modulus [20, 21], resistance to enzymatic degradation [22], and collagen fiber diameter [23]	Improvement in VA, K reading, refraction, and halt of ectasia progression

Epi-on CXL	Riboflavin penetration requires more time than with epi-off techniques Epithelium permeabilization can be achieved with molecules as cyclodextrins [24] and benzalkonium chloride in association with NaCl [25]	Improvement in VA and topographic findings Halt of ectasia progression There is a lot of controversy about results of this technique [26]

Ultrafast CXL	Young's modulus similar to traditional CXL [27]	Equivalent in VA, refraction and pentacam parameters [28, 29], and OCT imaging [29]

Athens protocol	No data available	Superiorly with a better BSCVA, mean K reduction, spherical equivalent, and corneal haza score [30]
